# Pharmacy Contributions to Improved Population Health: Expanding the Public Health Roundtable

**DOI:** 10.5888/pcd17.200350

**Published:** 2020-09-24

**Authors:** Mark A. Strand, Natalie A. DiPietro Mager, Lori Hall, Sarah Levin Martin, Daniel F. Sarpong

**Affiliations:** 1 School of Pharmacy College of Health Professions, North Dakota State University, Fargo, North Dakota; 2 Raabe College of Pharmacy, Ohio Northern University, Ada, Ohio; 3 Division of Strategic National Stockpile, Office of the Assistant Secretary for Preparedness and Response, Atlanta, Georgia; 4 Department of Community Health, University of Maine at Farmington, Farmington, Maine; 5 Center for Minority Health and Health Disparities Research and Education, College of Pharmacy, Xavier University of Louisiana, New Orleans, Louisiana

Success in health care is increasingly being measured by improvements in population health outcomes in response to interventions rather than by services delivered (1). In this new landscape, cross-sectoral collaboration is paramount (2), and the profession of pharmacy is an often-overlooked partner (3). Our vision is that pharmacists and the profession of pharmacy be included as an integral part of the roundtable of health care and public health. This special collection of articles in *Preventing Chronic Disease* highlights the contributions of pharmacy to the field of public health and expands the vision of how pharmacy can improve population health.

The collection brings together some of the cutting-edge work at the interface of pharmacy and public health that was submitted in response to a call for papers in 2019. The Centers for Disease Control and Prevention (CDC) has long recognized pharmacy’s role in addressing chronic diseases (4). The 14 articles included in this collection document a small portion of the innovative work being done by pharmacists to improve population health. We first review the articles to summarize research approaches and contributions. We then describe gaps in research that need to be filled to strengthen the evidence base for the unique role of pharmacy in improving population health. The collection serves as a call to researchers and professionals in pharmacy and public health to evaluate and publish their work in hopes of expanding on what is already known and being done.

## Collaboration of Pharmacy With Other Health Care Agencies

Collaboration between pharmacy and other health care professionals and agencies to implement strategies to improve health outcomes is well represented in this collection (5–8). Articles by Rodis et al (5) and Ross et al (6) highlight the impact of pharmacists providing collaborative medication therapy management services to patients in federally qualified health centers. In another description of a collaborative model for medication therapy management implemented by the Pharmacy Society of Wisconsin, the Wisconsin Division of Public Health, and a nonprofit insurer, Thompson et al showed improvements in self-reported use of self-management tools, reductions in medication adherence barriers, and high levels of satisfaction with the pharmacist in controlling hypertension (7). Collaboration between the New Mexico Department of Health and community pharmacies demonstrated the ability of community pharmacists to safely administer latent tuberculosis treatment, with a satisfactory completion rate of 75.0% (8). The program was implemented in collaboration with the local public health department, thus saving time for their health care providers. Strand et al reported on the many ways in which community pharmacy has responded to the coronavirus disease 2019 (COVID-19) pandemic, with recommendations for deepening formal collaboration with local public health agencies (9). Sun et al reported on pharmacy students training high school students about opioid misuse (10), showing the opportunity for collaboration with public schools. The integration of pharmacy with clinical medicine has been recommended by CDC (4) and the American College of Cardiology (11), and these publications demonstrate that integration with other health care agencies and the community lead to improved health outcomes.

Many Americans do not receive the services recommended by the US Preventive Services Task Force (USPSTF) (12). Although community and clinical pharmacists could be key players in delivering these services (13), barriers to pharmacies receiving reimbursement for the delivery of some of these services compromises the full incorporation of the delivery of USPSTF services into many community pharmacy settings (14). Several articles in this collection speak to this problem. In 2018, only 51.1% of US adolescents aged 13 to 17 were fully covered by the human papillomavirus (HPV) vaccine series (15), so the need for health care providers other than physicians to administer HPV vaccines in various settings is great. Yet as Ryan et al found, both pharmacists and community members identified more barriers than facilitators to providing and receiving the HPV vaccine in the pharmacy setting (16). Work is needed to determine best practices for removing barriers that prevent community pharmacists from delivering vaccines, especially as we anticipate a vaccine against severe acute respiratory coronavirus 2 (SARS-CoV-2). Freeland and Ventricelli made a call to pharmacists to promote the hepatitis B vaccine more aggressively among at-risk patients in settings heavily affected by the opioid epidemic (17). Clearly, the need exists to elevate both the self-efficacy of pharmacists in delivering all vaccines and the awareness among the general public about the appropriateness of pharmacists administering vaccines, to expand beyond vaccines that are currently most frequently administered — influenza, pneumococcal, and herpes zoster (shingles) vaccines.

Diabetes and its determinants are epidemic in the United States, and prevention and management are of vital importance (18). To that end, Roszak and Ferreri conducted key informant interviews among pharmacy executives to identify barriers to and opportunities for implementing the National Diabetes Prevention Program (DPP) in community pharmacies (19). They concluded that realizing this opportunity will require reimbursement for pharmacists’ efforts, minimal disruption of routine workflow, and understanding among patients that pharmacists can provide this program effectively. Demonstrating that implementation of the DPP in community pharmacies is possible, Ross et al reported on the ability to nearly triple the number of pharmacies delivering the National Diabetes Prevention Program by following a systematic process and including stakeholders every step of the way (20). Pharmacies are present in most communities around the country, and patients have more interaction with their pharmacist than with any other health care providers (21–23), so pharmacists are well positioned to deliver preventive services. Delivery of USPSTF-recommended and other preventive health services should be expanded in community pharmacies to broaden the base of preventive service delivery across the population, but barriers remain to scaling up the delivery of these services by pharmacists. Widespread implementation of such services, with rigorous evaluation, is needed.

## Pharmacy Contributions to Improving Population Health

Since the seminal work of the Asheville Project demonstrated the effect of clinical pharmacists on improving outcomes in diabetes, hypertension, and hyperlipidemia in 2003 (24,25), pharmacist participation in care coordination to manage chronic conditions has been consistently demonstrated. Cowart et al described how a physician–pharmacist team brought a cohort of patients with diabetes to the hemoglobin A_1c_ goal of less than 7.0% in 99 fewer days than the usual medical care of physician alone (26). Clearly, a pharmacist brings added value to the care team.

In this era of health care workforce shortages across the country, pharmacists fill this gap by serving as critical members of team-based care. Two articles in this collection examined the question What happens when access to pharmacies is limited? Using claims data, Pathak et al showed that medication adherence among people with diabetes and hypertension using telepharmacy support was not inferior to medication adherence achieved in face-to-face support (27). Telepharmacy support creates opportunities to expand services to remote areas that lack an onsite pharmacist. Working in Washington State, Graves et al showed that the likelihood of access to a Medicaid-contracted pharmacy decreased significantly as rurality increased (28). To ensure medication access and adherence among low-income Americans who live in rural areas, rural pharmacies need to increase enrollment in Medicaid service provision.

Multisector collaboration is needed to address the epidemic of chronic diseases in the United States. Most chronic diseases depend on the use of long-term medications and high levels of adherence for successful management. As medication experts, the pharmacist is a natural member of the chronic disease management team. Studying US states and census regions, Yang et al found that prescription- and payment-related promoters of adherence to blood pressure medication varied by geography and across the largest patient market segments (medication prescriber, insurance payer type, and age) (29). Blood pressure control rates nationwide are inadequate and could be improved by uptake of promoter strategies such as fixed-dose combinations, mail order refills, being under the management of a designated primary care provider, and having commercial insurance. Many of these promoter strategies can be manipulated by pharmacists. More consistent use of these promoter strategies could increase adherence to blood pressure medication, but more consistent use requires incorporating pharmacists into collaborations that include prescription benefit manager programs, payers, and health care providers.

Evidence of pharmacists evolving beyond their traditional roles is apparent throughout CDC. Through numerous cooperative agreements, CDC’s National Center for Chronic Disease Prevention and Health Promotion instructs state health department grantees to engage pharmacists as health care extenders and in team-based care approaches (30). CDC recognizes pharmacists can help to achieve public health outcomes not only in chronic diseases but also in HIV testing, antimicrobial stewardship programs, immunizations, and many others. The role of pharmacists has come a long way, from dispensing, to providing clinical care, to now administering vaccinations, screening for diseases, and health coaching. They are, indeed, critical members of the public health roundtable.

## More Research Needed

Several important areas of research at the interface of pharmacy and public health were not covered in this collection. We now turn our attention to research areas that merit further evaluation and reporting.

Social determinants of health such as poverty, unequal access to health care and education, and racism are drivers of health inequities and, thus, are central to the public health mission to achieve health for all. Healthy People 2020 calls for approaches that address these social factors to help improve health equity for populations who are disproportionately affected by chronic conditions and other causes of death and disability (31). The pharmacy profession is sensitive to the social determinants of health: it prioritizes customizing patient care, a concern for cultural competency (32), and attention to health literacy (33), and it fosters each of these concepts through curricula and workforce development. For example, results from the Project IMPACT study show that pharmacists have improved health outcomes for diverse populations disproportionately affected by diabetes (34).

However, achieving health equity will require that social determinants of health be considered not only in how one treats an individual patient but also in the delivery of pharmacist-provided services more broadly, such as determining who receives care and how it is received. In a systematic review of 157 studies on public health services delivered by community pharmacists, none discussed health inequities (13). Qato et al showed that residents of predominantly low-income racial/ethnic minority communities on the south side of Chicago could not use their nearest pharmacy because of cost issues and had to travel further from home to overcome these issues (35); however, more research is needed on how social determinants can be integrated into delivery of care and the outcomes associated with their integration.

Another area of future research is training pharmacists to increase their public health skills to improve population health beyond traditional pharmacy functions. The number of doctor of pharmacy/master of public health (PharmD/MPH) dual degree programs is increasing (36), but enrollment in these programs is not high. Although pharmacy education accreditation standards related to public health competencies exist (37), many schools of pharmacy do not prioritize public health competencies in their curricula. Postgraduate training in public health competencies is another way of conceptualizing public health education for pharmacists. One such example is a 3-hour continuing education training program for pharmacists to implement screening of opioid misuse in community pharmacies (38). The researchers showed improvement in the attitudes and perceptions among pharmacists about opioid-related patient behaviors and the clinical value of screening for opioid misuse. It would be helpful to know what further public health education pharmacists need, and which types of training directly lead to improved population health.

Being located in the community and having the most frequent interaction with patients, compared with all other health professionals (21,22), pharmacists could collaborate with public health to identify and implement systems for disease surveillance and monitoring health outcomes (39). Such systems represent another research gap in this collection, but not entirely. Matus et al used GIS mapping to track opioid use in wastewater, stating, “These maps can in turn provide an evidentiary basis for deployment of pharmacy-centered public health responses” (40). A search of the literature provides further examples. A unique system in Maine used public records from law enforcement to inform medical providers of potential misuse and diversion of narcotic medication (41). Another example of surveillance by community pharmacies is Walgreens’ use of Esri location analytics to track retail prescription data for antiviral medications used to treat influenza (42). The volume of antiviral medications dispensed serves as a proxy for the temporal and geographic spread of the influenza season in real time. Linked to the local epidemiology division of the public health department, the data generated by sales records of antiviral medication could lead to early mitigation of influenza outbreaks. However, this linkage would require formal integration of pharmacy and public health informatics systems, something that still needs to be improved.

In a systematic review of 522 studies on the contributions of pharmacy to the 10 essential services of public health, the 2 services least represented were community health needs assessments and diagnosing health problems in the community (43). Community health needs assessments are a key element of the Affordable Care Act and have increased engagement of hospitals in the communities they serve. However, little published evidence exists of pharmacies collaborating with hospitals and public health agencies to conduct these needs assessments. Although some might argue that such work is outside the areas of training for pharmacists, the community location of pharmacists and their accessibility to populations gives pharmacists a unique opportunity to participate in community health needs assessments. None of the articles in this *Preventing Chronic Disease* collection reported on this area of research. Furthermore, a PubMed search identified 26 studies on community health needs assessments, but none of these studies included pharmacy. We see this gap as an opportunity to expand the viewpoint of community health needs assessments and increase access to community members to better inform needs assessments.

We recognize a need for clear criteria by which to evaluate pharmacist contributions to intervention studies (44). Several aspects of interventions should be evaluated (45), such as whether the intervention was implemented as intended as well as its effectiveness and cost-effectiveness. Many studies have shown the effectiveness of pharmacy services as measured by patient outcomes or cost-effectiveness (46), but process evaluations are scarce, especially for services that demonstrate collaboration between public health agencies and pharmacists. Process evaluations involve critical appraisal of whether the intended activities are taking place, who is performing the activities, who is affected by the activities, and whether sufficient resources have been allocated to accomplish the purpose of the intervention (44,45). Evaluations should be performed in such a way that they determine the unique attributes and distinct value provided by collaborations that include pharmacy partners as compared with collaborations that include other disciplines. Additionally, the plan for evaluation should begin while the program is being designed (45). Many readers of *Preventing Chronic Disease* are familiar with such models as RE-AIM (Reach, Effectiveness, Adoption, Implementation, Maintenance) (47), and this model has been used to evaluate the population impact of projects implemented in community pharmacies (48). Such evaluation tools, considered best practices in public health, need to be more frequently implemented in pharmacy interventions (49).

It is evident from the small sample of studies articulating the contributions of pharmacists or pharmacies in addressing the health of the population that much work is yet to be done. The pharmacy profession has made advances and contributions, but gaps in service exist and the role of pharmacists in public health needs to be broadened. This recognition leads us to a call to action by both pharmacy and the public health professions to expand their collaboration to improve population health and mitigate health inequities.

## Call to Action

The health care system, including pharmacy and public health, have opportunities to improve population health through greater collaboration (13). To realize this opportunity, partnerships need to be strengthened, current barriers need to be removed, and pharmacists need to be more fully integrated into community health needs assessments, disease surveillance, and monitoring of health outcomes. Furthermore, the profession of pharmacy needs to become more proactive in pursuing opportunities to make these contributions, evaluate them, and then publicly report on them.

Leaders in public health and pharmacy should develop more partnerships that serve to mutually benefit each sector’s goals and leverage their strengths. Readers of this collection will find many examples of public health partnering with pharmacists to deliver their programs at the federal, state, and local levels. Pharmacists are uniquely positioned to enhance the quality, reach, and sustainability of preventive services. As pharmacists are asked to implement more preventive services, public health partners have opportunities to apply their expertise to support them, thus establishing mutually beneficial collaborations. For example, public health partners can help pharmacists evaluate their process and outcomes to strengthen the way they capture and communicate success stories, especially to nonpharmacist audiences. Public health partners should be more proactive in ensuring that pharmacy representatives are a part of statewide health planning efforts. Public health and pharmacy leaders can also advocate for policies that reduce the current obstacles to pharmacists delivering preventive and health promotion services.

Barriers need to be considered, with interventions designed to overcome those barriers. Currently, privileges granted to pharmacists in most states do not ascend to the level of their training. An article in this collection by Hamilton et al raises awareness of this issue by describing barriers in Louisiana (50). All states need to grant pharmacists privileges to practice at a level commensurate with their training and education and require third-party payers to reimburse pharmacists for their services. These steps are necessary to fill shortages in the primary care workforce and enable pharmacists to contribute more substantially to improved population health.

The public health infrastructure needs to use pharmacists better in community health needs assessments, disease surveillance, and monitoring of health outcomes. This infrastructure improvement will require transformation in what data pharmacists have access to and contribute to. Community pharmacies now exist in a patient-information vacuum. We need to break down the barriers that isolate community pharmacy from the wider public health and health care systems and to include pharmacy in health information exchanges and surveillance systems.

In addition, the pharmacy profession must aggressively pursue the opportunities available to it. Collaborations with local health care entities in community-based health interventions could be expanded. Pharmacists will need to envision themselves as participants in the wider community and seek ways to collaborate with other health care professions. More collaboration could be achieved in part by pharmacists stepping out of their comfort zone and welcoming people from various disciplines into their professional organizations and meetings. The pursuit of new opportunities will also require expanded training in public health. Although the PharmD degree affords a high level of training in patient care and medication management, it has competency gaps in public health skills such as informatics, program design and evaluation, and policy development. Finally, pharmacists needs to advocate more proactively for their role in the public health arena and to raise awareness of their contributions by publishing more often in journals read by a wider audience than just pharmacy researchers.

We hope that this collection of articles in *Preventing Chronic Disease* will spur others involved in improving population health through pharmacy applications to share their work and expand their research in this arena. Dissemination of information on the contribution of the pharmacy profession to public health is essential to creating awareness among other health professionals and the public about the integral role of pharmacy in public health. Such awareness is crucial to addressing health disparities, given that in most underserved communities, pharmacies are the initial point of contact with the health infrastructure. To this end, we advocate for more integrated involvement of pharmacists in public health and the dissemination of information on their contributions to the health of the people.
